# Trauma-focused treatment for posttraumatic stress disorder combined with CBT for severe substance use disorder: a randomized controlled trial

**DOI:** 10.1186/1471-244X-13-172

**Published:** 2013-06-19

**Authors:** Debora van Dam, Thomas Ehring, Ellen Vedel, Paul MG Emmelkamp

**Affiliations:** 1 Department of Clinical Psychology, University of Amsterdam, Weesperplein 4, 1018 XA, Amsterdam, The Netherlands; 2 Jellinek Substance Abuse Treatment Center, Arkin, Postbus 3907, 1001 AS Amsterdam, The Netherlands; 3 Institute of Psychology, University of Münster, Fliednerstr. 21, 48149, Münster, Germany; 4 King Abdulaziz University, P.O Box 80203, Jeddah, Saudi Arabia

**Keywords:** Posttraumatic stress disorder, Substance use disorder, Comorbidity, Randomized controlled trial, Trauma-focused treatment, Integrated treatment

## Abstract

**Background:**

This randomized controlled trial (RCT) investigated the effectiveness of a combined treatment for co-morbid Posttraumatic Stress Disorder (PTSD) and severe Substance Use Disorder (SUD).

**Methods:**

Structured Writing Therapy for PTSD (SWT), an evidence-based traumafocused intervention, was added on to Treatment As Usual (TAU), consisting of an intensive cognitive behavioral inpatient or day group treatment for SUD. The outcomes of the combined treatment (TAU + SWT) were compared to TAU alone in a sample of 34 patients.

**Results:**

Results showed a general reduction of SUD symptoms for both TAU + SWT and TAU. Treatment superiority of TAU + SWT was neither confirmed by interaction effects (time x condition) for SUD or PTSD symptoms, nor by a group difference for SUD diagnostic status at post-treatment. However, planned contrasts revealed that improvements for PTSD severity over time were only significant within the TAU + SWT group. In addition, within the TAU + SWT group the remission of PTSD diagnoses after treatment was significant, which was not the case for TAU. Finally, at post-treatment a trend was noticed for between group differences for the number of PTSD diagnoses favoring TAU + SWT above TAU.

**Conclusions:**

In sum, the current study provides preliminary evidence that adding a trauma-focused treatment on to standard SUD treatment may be beneficial.

## Background

Over the past decade, the detection and treatment of posttraumatic stress disorder (PTSD) among substance use disorder (SUD) patients have increasingly been studied [1-3]. This trend mirrors a need in clinical practice as the number of SUD patients meeting diagnostic criteria for PTSD is relatively large (20-30%) [2,3]. Importantly, there is evidence that this patient group suffers from more severe complaints and more relapses in substance use than SUD patients without comorbid PTSD [4,5]. This suggests that the common treatment approach, whereby SUD and PTSD are treated sequentially and within different treatment centers, may not be optimal [6-8].

Several theories have been developed to explain the high comorbidity between PTSD and SUD. Most evidence is available for the self-medication theory [9], which suggests that substances are used to alleviate or suppress PTSD symptoms. In line with this theory, research investigating the chronology of PTSD and SUD has shown that SUD is preceded by PTSD more often than vice versa [10,11], that the exacerbation of PTSD symptoms is the most important factor in predicting relapse following SUD treatment [12], and that improvements in PTSD symptoms are associated with subsequent improvements in substance dependence [13,14]. In addition, experimental research suggests that trauma-related cues can trigger a craving response [15].

On the other hand, there are also theoretical and empirical grounds to assume an inverse relationship. The high risk hypothesis poses that SUD augments the risk for traumatic experiences and thereby the chance for developing PTSD [16]. In addition, SUD may interfere with habituation to the trauma memory [11], and the withdrawal of substances may evoke traumatic memories and trigger PTSD symptoms as it resembles physical experiences during trauma [11]. In line with these hypotheses, there is some evidence to suggest that in some cases SUD precedes PTSD in the development of this comorbidity; in addition, the treatment of SUD alone has been shown to lead to a reduction of PTSD symptoms [17-20].

Taken the two perspectives together, a reciprocal relationship between both disorders appears to be the most likely explanation for the high co-morbidity between PTSD and SUD [1,11]. This hypothesis is also supported by recent data indicating that the vast majority of patients first reported trauma, then substance use, which again was followed by additional traumatic experiences, and further substance use [21]. This chronology suggests that patients’ substance use indeed increases after having experienced trauma, and that high levels of substance use may in turn increase the risk for other traumatic events. A mutual relationship between PTSD and SUD implies that PTSD symptoms may exacerbate in the first period of abstinence, and that PTSD complaints may improve when abstinence is maintained. Consequently, it appears likely that a sequential treatment approach increases the risk that patients drop out of SUD treatment prematurely and therefore do not receive PTSD treatment either. It is therefore plausible to assume that patients will benefit more from combined treatment interventions for PTSD and SUD.

Existing treatments for concurrent PTSD and SUD are based on two different approaches. Some authors suggest that PTSD among SUD patients should be treated according to the guidelines for PTSD in general, which recommend trauma focused-cognitive behavioral treatment (TF-CBT) and EMDR [22]. An important element of TF-CBT is imaginal exposure. Patients are asked to revisit their traumatic event in their imagination and describe it in great detail [23]. In EMDR, the client is instructed to focus on the traumatic memory and simultaneously perform rhythmic eye movements [24].

A contrasting point of view is that trauma-focused interventions may be too invasive for patients with concurrent PTSD and SUD, and that these interventions put patients at risk for relapse, treatment dropout and other adverse events [25,26]. Based on this idea, non-trauma-focused interventions have been developed that focus on the present or past aspects of the patient’s life other than the trauma, and that do not require patients to revisit or reprocess the trauma [e.g. 25]. The aim of these treatments is to provide patients with coping skills to manage their trauma symptoms and to improve functioning. The majority of treatments developed for concurrent PTSD and SUD to date are non-trauma-focused [7,27-29]. Although some programs include in vivo exposure [29] or sharing traumatic experiences within the group [28], they are best characterized as non-trauma-focused treatments as they do not comprise exposure to the trauma memory as a main ingredient. Existing integrated treatments using a non-trauma-focused approach appear to be successful in reducing PTSD and SUD symptoms, but their results are generally not superior to active control conditions, such as regular SUD treatment [1,30,31]. However, integrated cognitive behavioral therapy, a non-trauma-focused therapy based on a cognitive restructuring approach, appears to be a positive exception to this rule [21].

Recent evidence suggests that patients with concurrent PTSD and SUD may benefit from trauma-focused interventions, and that these interventions are more effective in reducing symptoms of PTSD than treatment-as-usual [1,32,33]. Importantly, it appears that exposure-based interventions are not necessarily associated with an increase in attrition or relapse to drugs or alcohol [35]. Until now, trauma-focused interventions have not been studied within severe SUD patients allocated to intensive SUD treatment. Attention for PTSD symptoms appears especially important for this patient group as untreated PTSD symptoms can be expected to be related to a number of clinical complications. Earlier research has shown that PTSD symptoms in SUD patients are associated with increased relapse in substance use [13,35,36], and with more problems in mental health, physical health, and social relationships [37]. To our knowledge, this randomized controlled trial (RCT) is the first study bridging this gap.

An evidence-based trauma-focused intervention for PTSD was added on to a regular intensive cognitive behavioral SUD program for severe SUD patients, which was the treatment-as-usual (TAU) for this sample [38]. The study aimed to investigate the effectiveness of adding PTSD treatment to the intensive SUD treatment program compared to TAU, i.e. the intensive SUD treatment program only. The trauma-focused intervention was Structured Writing Therapy (SWT) for PTSD [39]. SWT uses specific writing assignments to reprocess painful trauma memories, and it encourages cognitive reappraisal of trauma-related thoughts and social sharing of the traumatic event. Results from several studies support the effectiveness of SWT in the treatment of PTSD [39-41]. In addition, SWT has been shown to reduce levels of intrusions and avoidance, depression, anxiety and somatization [41].

The current study originated from an RCT investigating the effectiveness of an *integrated* outpatient treatment for concurrent PTSD and SUD (Van Dam, Vedel, Ehring, Emmelkamp: Integrated trauma-focused treatment for concurrent posttraumatic stress disorder and substance use disorder: a randomized controlled trial, submitted). In comparison to the earlier study, the current investigation focused on patients with more severe SUD symptoms who were attending inpatient or day treatment. Furthermore, in contrast to the earlier study among outpatients SWT was not integrated into the SUD intervention, but *added on* to TAU. The patients randomized to the experimental condition (TAU + SWT) received 10 individual sessions of SWT in addition to the regular SUD program. An *add-on* approach seemed more appropriate for this study as SWT is provided as an individual therapy. By adding SWT on to the regular SUD program, all patients received the same group intervention for SUD. Therefore, they all benefited equally from group dynamics, and they all received the same dose of SUD treatment whether they were allocated to TAU or TAU + SWT.

The aim of this RCT was to investigate the effectiveness of a combined treatment for comorbid PTSD and severe SUD. Three hypotheses were tested. The first hypothesis was based on the theory that PTSD and SUD are reciprocally related. In line with this assumption, we expected that both TAU and TAU + SWT would be effective in decreasing symptoms of SUD and PTSD. Secondly, we expected that patients receiving TAU + SWT would achieve significantly higher improvements on PTSD symptoms than patients in the TAU condition. Thirdly, following from the self-medication hypothesis we hypothesized that TAU + SWT would be more effective in reducing symptoms of SUD than TAU alone.

## Method

### Participants

**Figure 1:**
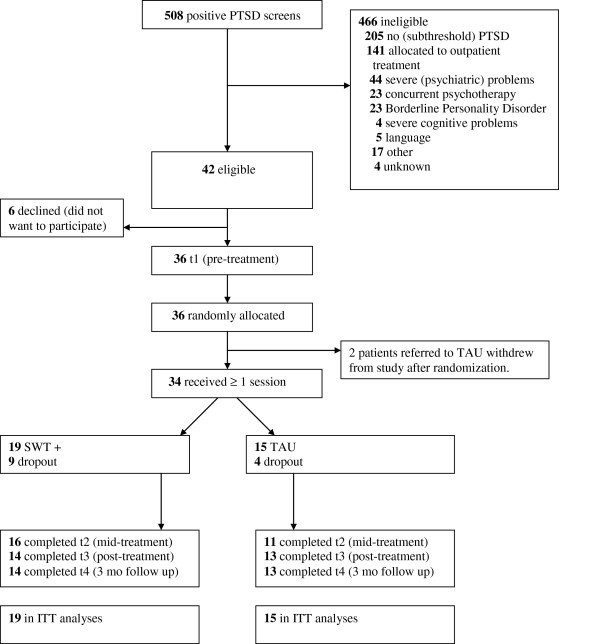
**CONSORT flowchart of the recruitment and retention of participants.** t1 = baseline; ITT = Intent-to-treat.

Figure 1 summarizes the flow of participants through the study. A consecutive sample of 34 patients was recruited from the Jellinek Substance Abuse Treatment Center in Amsterdam, The Netherlands. All patients were allocated to intensive inpatient or day group treatment for SUD. Allocation for treatment followed the principles of stepped care. Therefore, all patients included in the current study suffered from severe substance abuse, and had already been allocated to two or more SUD therapies in the past five years. Three patients dropping out the earlier study investigating *integrated* outpatient treatment for concurrent PTSD and SUD (Van Dam, Vedel, Ehring, Emmelkamp: Integrated trauma-focused treatment for concurrent posttraumatic stress disorder and substance use disorder: a randomized controlled trial, submitted) were also included into the current study. Recruitment and eligibility criteria were parallel to this earlier study. Patients were recruited between July 2008 and July 2011. Inclusion criteria were: (1) a diagnosis of substance abuse or substance dependence according to DSM-IV, (2) a diagnosis of full-blown or partial PTSD [42] according to DSM-IV (partial PTSD was defined as meeting symptom criteria for the reexperiencing cluster and for either the avoidance/numbing cluster or the hyperarousal cluster), (3) being allocated to intensive group treatment either as day treatment or as in-patient, (4) being 18 years or older, and (5) sufficient understanding of the Dutch or English language. Exclusion criteria were (1) a diagnosis of Borderline Personality Disorder, (2) other severe (psychiatric) problems that required immediate clinical care (e.g., psychotic symptoms, manic episode, current suicidal ideation, severe domestic violence), (3) severe cognitive disorders, or (4) receiving concurrent psychotherapy for any kind of psychological disorder. Patients receiving medication for psychological complaints (e.g., antidepressant medication) were included in the study if they remained on a stable dose during the course of the study. This was the case for six patients (18%). At 3 month follow-up, patients were asked whether they had been any change in medication prescription during the follow-up interval. One patient (3%) reported a change in medication treatment between post-treatment and follow-up, and two patients (6%) reported to have started new medication treatment within a month after treatment. No group differences were found between the CBT/SUD + SWT and CBT/SUD condition for the number of patients using medication during treatment, or medication changes after treatment (Fisher’s exact test; *p’s* > .245).

Patients in both conditions were considered dropouts if they ended TAU for SUD prematurely. Patients in the TAU + SWT group were additionally labeled as dropout if they attended less than 75% of the SWT treatment sessions (≤ 7). Dropout patterns for TAU + SWT (*N* = 19) revealed that ten patients completed treatment (53%). The other nine patients ended treatment before the fifth SWT session (47%). Three of them dropped out of treatment even before SWT started (33%). In this study, non-response was not equal to treatment dropout, as all patients could participate in study measurements whether they completed treatment or not.

Tables 1 and 2 summarize sample characteristics and between group analyses. The overall sample consisted of 23 males and 11 females, with a mean age of 42.3 (*SD* = 9.0). No significant differences between treatment conditions were found for sample characteristics, or dropout rates *χ*^*2*^*’s* (1, *N* = 34) *≤* 3.03, *p’s* ≥ .22. In addition, no group differences were revealed for baseline symptom severities *t*’s (32) *≤* 0.617, *p’s* ≥ .54, or for diagnostic status, Fisher’s exact *p’s* ≥ .08.

**Table 1:** Sample characteristics: demographic variables

**Demographics**	**Total ( *n = 34 *)**	**TAU + SWT ( *n = 19 *)**	**TAU ( *n = 15 *)**	**Between group analyses**
**Mean age (SD)**	42.3 (9.0)	42.6 (8.4)	41.9 (10.0)	t (33) = 0.21, *p* = .84
**Gender, *n *(%)**				χ^2^ (1, N = 34) = 0.012, *p* = .92
Male	23 (67.6)	13 (68.4)	10 (66.7)	
Female	11 (32.4)	6 (31.6)	5 (33.3)	
**Ethnicity, *n *(%)**				χ^2^ (4, N = 34) = 2.962, *p* = .56
Dutch	23 (67.6)	13 (68.4)	10 (66.7)	
European (other)	2 (5.9)	1 (5.3)	1 (6.7)	
Arabic/ Moroccan/ Turkish	4 (11.8)	1 (5.3)	3 (20.0)	
Black/ Surinamese/ Caribbean	4 (11.8)	3 (15.8)	1 (6.7)	
Other	1 (2.9)	1 (5.3)	0 (0)	
**Education (certificate), *n *(%)**				χ^2^ (3, N = 34) = 0.404, *p* = .94
No education, primary school	11 (32.4)	6 (31.6)	5 (33.3)	
Secondary school, lower level	8 (23.5)	4 (21.1)	4 (26.7)	
Secondary school, higher level	9 (26.5)	5 (26.3)	4 (26.7)	
Postsecondary	6 (17.6)	4 (21.1)	2 (13.3)	
**Relationship status, *n *(%)**				χ^2^ (2, N = 34) = 2.859, *p* = .24
Single	31 (91.2)	17 (89.5)	14 (93.3)	
Partner	2 (5.9)	2 (10.5)	0 (0)	
Missing	1 (2.9)	0 (0)	1 (6.7)	
**Source of income, *n *(%)**				χ^2^ (2, N = 34) = 3.026, *p* = .22
No work	22 (64.7)	10 (52.6)	12 (80.0)	
Work	11 (32.4)	8 (42.1)	3 (20.0)	
Missing	1 (2.9)	1 (5.3)	0 (0)	
**Dropouts, *n *(%)**				
SUD treatment & SWT	13 (38.2)	9 (47.4)	4 (26.7)	χ^2^ (1, N = 34) = 1.521, *p* = .30
SUD treatment	12 (35.3)	8 (42.1)	4 (26.7)	χ^2^ (1, N = 34) = 0.875, *p* = .48
Baseline Measures				
Mean PDS (SD)	29.5 (10.0)	30.4 (9.7)	28.3 (10.7)	t (33) = 0.62, *p* = .54
Mean TLFB (SD)	20.0 (27.2)	19.9 (29.3)	20.1 (25.4)	t (33) = 0.19, *p* = .99

*Note: TAU* Treatment As Usual, *SWT* Structured Writing Treatment.

**Table 2:** Sample characteristics: diagnostic status (current)

**Diagnostic status**	**Total ( *n = 34 *)**	**TAU + SWT ( *n = 19 *)**	**TAU ( *n = 15 *)**	**Between group analyses (Fisher’s exact)**
**PTSD diagnosis (full-blown), n (%)**	21 (61.8)	9 (47.4)	12 (80.0)	*p* = .08
**Primary SUD diagnosis, *n *(%)**				
Alcohol, not in remission	16 (44.1)	11 (57.9)	5 (33.3)	*p* = .19
Drugs, not in remission	15 (44.1)	8 (42.1)	7 (46.7)	
Cannabis	4 (11.8)	1 (5.3)	3 (20.0)	*p* = .30
Cocaine	10 (29.4)	6 (31.6)	4 (26.7)	*p* = 1.0
Other	1 (2.9)	1 (5.3)	0 (0)	*p* = 1.0
Substance Dependence	30 (88.2)	18 (94.7)	12 (80.0)	*p* = .30
Substance Abuse	1 (2.9)	1 (5.3)	0 (0)	*p* = 1.0
**Other axis-I diagnoses, n (%)**				
Depressive disorder	11 (32.4)	4 (21.1)	7 (46.7)	*p* = .15
Panic disorder	3 (8.8)	1 (5.3)	2 (13.3)	*p* = .57
Panic disorder with agoraphobia	2 (5.8)	0 (0)	2 (13.3)	*p* = .19
Social Phobia	4 (11.8)	2 (10.5)	2 (13.3)	*p* = 1.0
Specific phobia	2 (5.8)	1 (5.3)	1 (6.7)	*p* = 1.0
Obsessive compulsive disorder	0 (0)	0 (0)	0 (0)	-
General anxiety disorder	1 (2.9)	0 (0)	1 (6.7)	*p* = .44
Eating disorder	0 (0)	0 (0)	0 (0)	-

### Treatments

Treatment as usual (TAU) consisted of a regular intensive treatment program for SUD based on the principles of cognitive behavioral therapy (CBT) [38]. The treatment was delivered in a group format, and included coping skill training for alcohol and/or drug abuse, an evidence-based treatment for SUD [38]. Coping skill training for SUD teaches patients to recognize high risk situations preceding substance use, and offers strategies to deal with craving and relapse. Training tools are modeling, behavioral practice and homework assignments [43]. Coping skills training for SUD was offered twice a week for the first six weeks (2 h group sessions). After that, weekly sessions were provided for a period of 8 weeks. Furthermore, TAU incorporated social skills training, relaxation training, psycho-education, motivational interviewing sessions, basic CBT-training, relapse prevention sessions and emotion-regulation training. In addition to attending the group training program, patients had weekly sessions with an individual therapist. No interventions related to PTSD symptoms were carried out during these individual treatment sessions. The duration of the intensive part of the treatment program varied from 6 to 12 weeks. On average, patients attended the program four days a week. Dependent on the individual needs of each patient, TAU could be followed on an inpatient or an outpatient (day treatment) basis. All patients followed a detoxification program before starting the treatment program.

TAU + SWT existed of the same treatment program as described above, except for ten individual sessions of SWT that were added on to the program. SWT started after patients had been abstinent for 4 to 6 weeks. The treatment was drawn from a former protocol [44]. Therapy sessions were offered weekly and lasted 45–60 min. SWT consists of the following three phases: self-confrontation, cognitive reappraisal and sharing/farewell. The self-confrontation phase comprised trauma-focused exposure, and guided patients to write in detail about the most traumatic event(s) they had experienced. The writing had to be in the first person and in the present tense, addressing sensory experiences, painful facts, thoughts and emotions experienced during the trauma. The phase of cognitive reappraisal focused on changing dysfunctional appraisals related to the traumatic event and its consequences. For this purpose, patients were asked to write a letter of advice to an (imaginary) friend or loved one, imagining that they had experienced the same event. Patients were asked to give advice to this person on how to handle thoughts, emotions and consequences related to the trauma. In a second step, the patient was instructed to write a similar letter to him- or herself. The final phase consisted of a ‘sharing and farewell ritual’ that was aimed at finding closure of the traumatic event(s). In this final letter, the patient reflected on the trauma, its impact on his/her life, and his/her resolutions for dealing with the trauma in the future. During the whole treatment, writing assignments were introduced and discussed during the treatment sessions. TAU + SWT also incorporated two flexible sessions. Patients and therapists could decide what of the former SWT assignments they wanted to give extra attention. If necessary, it was possible to use the flexible sessions in advance to prolong the self-confrontation or the cognitive reappraisal phase.

In order to prepare patients with concurrent PTSD and SUD for possible difficulties during detoxification and SUD treatment, psycho-education about the vicious circle of PTSD and SUD was provided in the first treatment session [6]. For ethical reasons, psycho-education was not only provided in the TAU + SWT condition, but also in the TAU condition. Patients in the TAU + SWT group received psycho-education from their SWT therapist. In the TAU condition, psycho-education was provided by the individual TAU therapist.

### Therapists

All SWT therapists were regular therapist of the Jellinek with a master’s degree in clinical psychology and additional formal training in cognitive behavioral therapy. Therapist treatment adherence was monitored in weekly supervision sessions by the last author.

### Measures

The outcome measures for PTSD and SUD were change in the severity of PTSD symptoms and change in substance use, respectively. Further outcome measures were PTSD and SUD diagnostic status. The *Posttraumatic Diagnostic Scale (PDS)*[45] was used to assess PTSD symptom severity. The PDS consists of 17 items corresponding to the DSM-IV PTSD, that are rated on a 4-point Likert-scale (0 = not at all or only one time; 3 = five or more times a week/almost always), and 9 items assessing impairment in different life areas. PTSD symptom severity scores are obtained by summing the 17 symptom items, with higher scores indicating greater symptomatology [45]. The PDS has shown to perform well within an SUD population, revealing excellent internal consistency, good test –re-test reliability, and good convergent validity with PTSD diagnosis [46]. Also, high sensitivities, and moderate specificities were found for the PDS within this population [3,46]. By means of the *Timeline Follow Back (TLFB)*[47], retrospective estimates of daily use of alcohol and drugs were obtained for a time frame of 90 days. Its psychometric characteristics for alcohol use have been extensively evaluated [48,49]. In our study, alcohol consumption was converted to standard drinks, and drug use was converted to grams.

DSM-IV axis I disorders, including SUD and PTSD, were assessed with the *Structured Clinical Interview for DSM-IV Axis I Disorders (SCID-I)*[50,51]. The SCID-I has shown a fair interrater agreement for the SUD module (kappa = 0.65), and an excellent interrater agreement for the PTSD module (kappa = 0.77) [52]. To screen for (partial) PTSD, the *Jellinek-PTSD (J-PTSD)* screening questionnaire was used [53]. The J-PTSD was specifically developed to screen for PTSD in SUD patients. The sensitivity (.87), specificity (.75), and overall efficiency (.77) are high using a cutoff score of 2 [53]. The *McLean Screening Instrument for Borderline Personality Disorder (MSI-BPD)*[54] was used to screen for Borderline Personality Disorder (BPD). The MSI-BPD has shown a good sensitivity (.81) and specificity (.85) for a cutoff score of 7 [54]. Patients with a score of ≥ 7 were invited for further assessment. Borderline Personality Disorder was assessed with the *Structured Clinical Interview for DSM-IV Axis II Disorders (SCID-II)*[55]. The SCID-II has shown very high interrater agreement for the BPD module (kappa = 0.91) [52].

### Procedures

All patients attending a regular intake at the Jellinek were screened with the Jellinek-PTSD screening questionnaire for PTSD. If the screener was positive, patients were invited for further assessment in order to determine diagnostic status. If a formal diagnosis for (partial) PTSD was obtained, eligible patients received written information about the study and gave written informed consent. Patients willing to participate were invited again for the pre-treatment assessment (t1). After pre-treatment assessment, patients were randomly assigned to either TAU + SWT or TAU by asking them to draw one out of two closed envelopes. Each patient was approached for an additional three assessments during the study: mid-treatment (t2) (after the fifth session), post-treatment (t3), and 3 months post-treatment (t4). Patients were invited to the Jellinek treatment center for all assessments, except for the shorter 3-month follow-up, which was administered via telephone. If a patient was unable to come to the Jellinek for a face-to-face assessment, the mid-treatment or post-treatment assessments were also administered by telephone (*n* = 7 at post-treatment). There was no financial compensation for research and treatment participation.

The study was approved by the ethics committee of the University of Amsterdam (Faculty of Social and Behavioral Sciences; reference number 2008-KP-342), and submitted to the Clinical Trials Register, ClinicalTrials.gov (Trial # NCT00763542).

### Statistical methods

All analyses were performed using the IBM Statistical Package for Social Science (SPSS), version 19.0 for Windows. *T* tests and *χ*^*2*^ tests were used to compare both treatment conditions on sample characteristics and dropout rates. Treatment effects were investigated with intent to treat (ITT) analyses. Patients were categorized as ITT if they attended at least one therapy session. Overall missing data patterns showed very low percentages of item non-response (< 2%), except for one secondary outcome measure concerning craving (5% item non-response). This justified the use of response function imputation [56]. Figure 1 shows that unit non-response was lower than 21% over all measurements.

Missing data by unit non-response was handled by Multiple Imputation (MI) (m = 5). Whole scales were imputed using the complete datafile (k = 91) [57]. All analyses were performed on average values derived from the imputed dataset. For the dependent variables PDS total score and TLFB (days of abstinence) a General Linear Model (GLM) repeated measures was performed. Planned repeated contrasts for time were performed for each condition separately (at mid-treatment, post-treatment, and at 3 month follow up). Rank-transformation was performed additionally if variables were not normally distributed [58].

Non-parametric tests were used to examine differences for diagnostic status (Fisher’s exact test, and McNemars χ^2^) from pre- to post-treatment and follow-up. Effect sizes were calculated for all primary outcome measures.

## Results

### Treatment effects

Descriptive data for the primary outcome measures are displayed in Tables 3 and 4. All values are estimated values based on pooled outcomes on the imputed dataset. The outcome measures for PTSD were PTSD symptom severity (PDS) and PTSD diagnostic status (SCID diagnosis). The outcome measures for SUD were the number of abstinent days (TLFB) and SUD diagnostic status (SCID diagnosis).

**Table 3:** Descriptive analyses for PTSD for intent to treat sample (N = 34) (estimated values)

**Variable (primary outcome measures)**	**TAU + SWT (A) *(N = 19)***				**TAU (B) *(N = 15)***				**GLM**	***Partial η***^***2***^	**Contrast**	***Partial η***^***2***^	**GLM**	***Partial η***^***2***^	**GLM**	***partial bη***^***2***^
	**pre**	**mid**	**post**	**fu**	**pre**	**mid**	**post**	**fu**	**Time**	**Time**	**1 pre-mid**	**1 pre-mid**	**condition**	**condition**	Time * condition	Time * condition
											**2 mid-post**	**2 mid-post**				
											**3 post-fu**	**3 post-fu**				
**PDS total: *M (SD)***	30.4 (9.7)	28.2 (9.0)	17.6 (12.0)	23.5 (14.8)	28.3 (10.7)	26.5 (9.8)	24.3 (9.1)	21.7 (9.4)	A + B **	0.166	**1** A ns B ns	A 0.036 B 0.037	ns	0.000	ns	0.059
											**2** A * B ns	A 0.341 B 0.040				
											**3** A ns B ns	A 0.143 B 0.062				
									**McN χ**^**2**^	***OR ***^***3***^			**Fisher t **^**4**^	***φ***^***5***^		
**SCID (PTSD) *n *(%)**																
PTSD	19 (100)	-	9.8 (51.8)	-	15 (100)	-	13.2 (88.0)	*-*	A*	0			-	−0.390	-	-
									B	0						
Partial & Full-blown																
Partial PTSD	10 (52.6)	-	2.8 (14.7)	-	3 (20.0)	-	2.4 (16.0)	*-*	A*	0.1			-	−0.028	-	-
									B	0.8						
Full-blown PTSD	9 (47.4)	-	7 (36.8)	-	12 (80.0)	-	10.8 (72.0)	*-*	A	0.7			-	−0.353	-	-
									B	0.8						

*Note. PTSD* Posttraumatic Stress Disorder, *TAU* Treatment as usual, *SWT* Structured Writing Therapy, *PDS* Posttraumatic Diagnostic Scale, *SCID* Structured Clinical Interview of DSM IV, *PTSD* Posttraumatic stress disorder, A = TAU + SWT. B = TAU. *pre* pre-treatment, *mid* mid-treatment, *post* post-treatment, *Fu* follow up. McN χ^2^ = McNemars χ^2^. *OR* odds ratio, *Pl Con* planned contrasts.

*p <0.05 **p < 0.001. *t* trend, *ns* not significant.

^3^OR = increase of cases/ decrease of cases.

^4^Overall Fisher’s excact test was calculated for PTSD diagnosis (p = 0.06).

*φ*^*5*^ Phi (measure of effectsize).

**Table 4:** Descriptive analyses for SUD for intent to treat sample (N = 34) (estimated values)

**Variable (primary outcome measures)**	**TAU + SWT (A) *(N = 19)***			**TAU (B) *(N = 15)***			**GLM**	***Partial η***^***2***^	**Contrast**	***Partial η***^***2***^	**GLM**	***Partial η***^***2***^	**GLM**	***partial η***^***2***^
	**pre**	**post**	**fu**	**pre**	**post**	**fu**	**Time**	**Time**	**1 pre-post**	**1 pre-post**	**condition**	**condition**	Time * condition	Time * condition
									**2 post-fu**	**2 post-fu**				
**TLFB *M (SD) *Number of abstinent days**	19.9 (29.3)	76.8 (15.5)	61.0 (30.8)	20.1 (25.4)	66.0 (30.3)	58.6 (38.4)	A + B **	0.570	**1** A * B *	A 0.784 B 0.668	ns	0.011	ns	0.15
									**2** A * B ns	A 0.292 B 0.052				
							**McN χ**^**2**^	***OR ***^***5***^			**Fisher**	***φ***		
**SCID *n (*%) Primary SUD diagnosis**													-	-
In remission ^6^	1 (5.3)	16.6 (87.4)	-	3 (20.0)	10.2 (68.0)	-	A **	15.6/0			ns	−0.244	-	-
							B *	7						
**SCID *n *(%) **^**7 **^**Total N SUD diagnoses**													-	-
In remission	1 (5.3)	16.4 (86.3)	-	3 (20.0)	9.2 (61.3)	-	A **	15.4/0			ns	−0.293	-	-
							B t ^8^	4.9						
(Single SUD diagnosis, not in remission)	9 (47.4)	2.6 (13.7)	-	6 (40.0)	5.8 (68.7)	-	-	-			-	-	-	-
(2 SUD diagnoses not in remission)	6 (31.6)	0 (0)	-	3 (20.0)	0 (0)	-	-	-			-	-	-	-
(≥ 3 SUD diagnosesnot in remission)	3 (15.8)	0 (0)	-	3 (20.0)	0 (0)	-	-	-			-	-	-	-

*Note. PTSD* Posttraumatic Stress Disorder, *TAU* Treatment as usual, *SWT* Structured Writing Therapy, *PDS* Posttraumatic Diagnostic Scale, *SCID* Structured Clinical Interview of DSM IV, *PTSD* Posttraumatic stress disorder, A = TAU + SWT. B = TAU. *pre* pre-treatment, *mid* mid-treatment, *post* post-treatment, *Fu* follow up. McN χ^2^ = McNemars χ^2^. *OR* odds ratio, *Pl Con* = planned contrasts * p <0.05 **p < 0.001. *t* trend. *ns* not significant, *φ* Phi (measure of effectsize).

^5^OR = increase of cases/ decrease of cases (Breslow & Day, 1980). OR was not calculated when the denominator equals zero.

^6^SUD has been diagnosed, however the diagnosis is partially or completely in remission during the last month.

^7^No analyses were performed for variables in parentheses taking into account small sample sizes and inflation of chance.

^8^p = 0.06.

### PTSD symptom severity

GLM repeated measures analyses on the imputed dataset revealed a main effect for time, *F*(3, 34) = 6.37, *p* = .001, partial η^2^ = 0.166, and no main effect for condition *F*(1, 34) = 0.01, *p* = .921, partial η^2^ = 0. No significant interaction effect was found between condition and time *F*(3, 34) = 1.92, *p* = .132, partial η^2^ = 0.059.

Planned contrast analyses were performed for both treatment groups to assess the decrease in symptoms from pre- to mid-treatment, from mid- to post-treatment, and from post-treatment to follow-up. For the TAU + SWT group a significant decrease in PTSD severity was found from mid-treatment to post-treatment *F*(1, 19) = 9.31, *p* = .007, partial η^2^ = 0.341, but not from pre-treatment to mid-treatment *F*(1, 19) = 0.67, *p* = .424, partial η^2^ = 0.036, or from post-treatment to follow up *F*(1, 19) = 3.01, *p* = .100, partial η^2^ = 0.143. For the TAU group no significant decreases in PTSD symptom severity were found between the measurement points *F’s* (1, 15) ≤ 0.924, *p’s ≥* .353, partial η^2^’s ≤ 0.62.

### PTSD diagnostic status

For both conditions, differences for PTSD diagnostic status were investigated with McNemar χ^2^. Overall, a significant increase was found for the number of remitted cases (partial and full-blown) in the TAU + SWT condition, McNemars χ^2^ (1, *N* = 19) = 8.2, *p* = .004. More specifically, there was a significant decrease for partial PTSD McNemars χ^2^ (1, *N* = 19) =5.07, *p* = .024, but not for full-blown PTSD, McNemars χ^2^ (1, *N* = 19) = 0.17, *p* = .680. For TAU, no differences were found for PTSD diagnoses, McNemars χ^2^ (1, *N* = 26) ≤ 1.00, *p’s >* .317. To investigate differences for PTSD diagnoses between TAU + SWT and TAU at post-treatment, a Fisher’s exact test [a] was carried out. Post-treatment results indicated a trend for between-group differences in PTSD diagnostic status (*p =* .06). After TAU + SWT less patients were diagnosed with PTSD than after TAU.

### Abstinence

Overall abstinence from alcohol and drugs was calculated from participants’ TLFB reports for a 90 day time window. The GLM analysis showed a significant main effect for time *F*(2, 34) = 42.38, *p <* .001, partial η^2^ = 0.570, indicating an increase for the number of drug and alcohol free days from pre-treatment to follow up. Neither a main effect for condition *F*(2, 34) = 0.35, *p =* .557, partial η^2^ = 0.011, nor an Time x Condition interaction effect were found, *F*(2, 34) = 0.48, *p* = .620, partial η^2^ = 0.15. Outcomes were similar after rank-transformation [59].

For each treatment condition, planned contrast analyses were performed to assess changes in abstinence from pre- to post-treatment, and from post-treatment to follow-up. For TAU + SWT, significant increases in abstinence were found from pre-treatment to post-treatment *F*(1, 19) = 65.21, *p* < .001, partial η^2^ = 0.784, and significant decreases in abstinence from post-treatment to follow-up *F*(1, 19) = 7.422, *p* = .014, partial η^2^ = 0.292. The TAU group only revealed an increase for abstinence from pre-treatment to post-treatment *F*(1, 15) = 28.139, *p* < .001, partial η^2^ = 0.668. No significant changes for abstinence were found for TAU from post-treatment to follow-up *F*(1, 15) = 0.767, *p* = .396, partial η^2^ = 0.052.

### SUD diagnostic status

To compare SUD diagnostic status from pre- to post-treatment, McNemar χ^2^ analyses were performed for each treatment group. Outcomes for TAU + SWT all showed significant decreases for SUD diagnostic status. McNemars χ^2^’s (1, *N* = 19) ≥ 14.4, *p’s* < .001). For TAU, the number of Primary SUD diagnoses decreased significantly from pre- to post-treatment, McNemars χ^2^ (1, *N* = 15) = 4.7, *p* = .03, and a trend was noticed for the decrease of total number of SUD diagnoses, McNemars χ^2^ (1, *N* = 15) = 3.4, *p =* .06*.*

Post-treatment differences for SUD diagnostic status between TAU + SWT and TAU were investigated by means of Fisher’s exact tests [b] , which revealed no differences between both groups (*p’s >* .23).

## Discussion and conclusions

The aim of this RCT was to investigate the effectiveness of adding treatment for concurrent PTSD on to an intensive SUD treatment program. It was expected that the combination of these two evidence-based treatments would lead to improved prognoses.

According to the first hypothesis, a reduction of SUD and PTSD symptoms was expected in both conditions. This expectation was generally confirmed by findings for SUD. Overall, there was a significant decrease of SUD symptoms from pre-treatment to follow-up. Planned contrasts showed an increase in abstinence for both TAU and TAU + SWT during treatment, but also some decrease of improvements from post-treatment to follow-up for TAU + SWT. In addition, both groups showed a significant remission for the primary SUD diagnosis. Furthermore, a significant reduction for the total number of SUD diagnoses was found in the TAU + SWT group, and a trend was found for TAU. In sum, both conditions were effective in reducing SUD, which was to be expected as SUD was targeted in the same way in both groups. Importantly, the current results also show that it appears safe to provide trauma-focused treatment for PTSD in combination with SUD treatment, which is in contrast to frequent clinical belief.

Based on the idea that SUD and PTSD are mutually maintained by a vicious cycle, it was expected that successful SUD treatment should also reduce symptom levels of PTSD. Hypothesis 1 therefore also predicted that PTSD should significantly be reduced in both treatment conditions. At the same time, Hypothesis 2 predicted that PTSD should improve more after combination treatment compared to TAU. Analyses testing these two hypotheses provided somewhat mixed results. Symptom levels of PTSD significantly decreased over time in the overall sample, which can be interpreted as support for Hypothesis 1. In contrast to Hypothesis 2, no significant interaction between time and condition emerged, i.e. we did not find clear-cut evidence for a superiority of SWT + TAU over TAU. However, there was indirect evidence suggesting that the addition of SWT to TAU may be beneficial. First, planned contrasts showed only a significant reduction of PTSD symptoms during SWT for the TAU + SWT group, but no significant reductions for PTSD during or after TAU. This indicates that the overall decrease of PTSD in both groups could mainly be attributed to the results of the SWT + TAU condition. Furthermore, PTSD diagnoses decreased in both conditions, but this reduction was only significant in the TAU + SWT condition. Finally, at post-treatment a trend was found for between-group differences for PTSD diagnostic status, indicating that fewer patients were diagnosed with PTSD (partial or full-blown) after TAU + SWT than after TAU.

Thirdly, we expected that TAU + SWT would be more effective in reducing symptoms of SUD than TAU alone. This prediction was based on the self-medication hypothesis, which suggests that successful PTSD treatment may lead to more sustainable abstinence as the need to self-medicate is reduced. This hypothesis was not supported by any type of analysis.

In sum, both treatments were found to be equally effective in treating SUD. We found preliminary evidence suggesting that SWT + TAU may be more effective in treating PTSD symptoms than TAU, although this was not supported by the crucial Time x Condition interaction on PTSD symptom severities, but only by a number of indirect findings. The fact that the interaction effect was not significant may be due to different factors. First, differences between both groups were difficult to detect, due to the small sample size and therefore reduced power. Another possibility is that the dose of SWT treatment was too low to realize significant improvements for PTSD symptoms. Interestingly, the reduction of diagnostic status in the combination condition was only significant for the partial PTSD group but not for patients with full-blown PTSD. This could also be interpreted as support for the idea that trauma survivors with SUD and high symptom levels of PTSD may need a higher dose of treatment. In any case, a replication of the findings in a larger sample is necessary before any firm conclusions can be drawn.

In an earlier study, we evaluated integrated trauma-focused SWT for PTSD and CBT for SUD within a larger sample of outpatients (*N =* 96) (Van Dam, Vedel, Ehring, Emmelkamp: Integrated trauma-focused treatment for concurrent posttraumatic stress disorder and substance use disorder: a randomized controlled trial, submitted). These outcomes showed that PTSD and SUD symptoms were treated effectively in both conditions. In addition, completer analyses favored trauma-focused integrated treatment above CBT for SUD in reducing PTSD symptom severity. Apart from sample size, there were other important differences in sample characteristics between the present and the previous study. Most importantly, the current patient sample was more severe. This was mirrored by the need for a more intensive treatment program for SUD complaints, but also in less mean abstinent days at baseline (20 versus 34) and slightly higher mean baseline scores for PTSD (30 versus 27). Sample characteristics for both studies showed that the current sample comprised relatively more men, more patients with a lower education, more patients without a relationship, and more patients without work. Although the present patient group was more severe, overall dropout percentages were lower (overall: 38%; TAU + SWT: 47%; TAU: 27%), compared to the previous study (overall: 46%; TAU + SWT: 51%; TAU: 40%), but also compared to other findings in this area of research [1]. Importantly, dropout percentages did not differ significantly between conditions, although a study comprising a larger sample size and therefore higher statistical power is necessary to provide conclusive evidence on this issue. Although the dropout rates observed in the current study are comparable to earlier research in this field, they are nevertheless far from satisfactory. Future research should aim at improving the acceptability of integrated treatments for PTSD and SUD. Notably, in the current study most patients dropped out before SWT started (33%), or during the first phase of self-confrontation of the SWT treatment (56%). Only one patient ended treatment just after the self-confrontation phase (11%). This suggests that patients were inclined to shudder from, or terminate during, the assignments comprising trauma-focused exposure. Future studies should explore whether a longer phase of preparation for trauma-focused treatment may increase the acceptability of this type of intervention.

The lack of significant between-group differences for SUD in the current study is consistent with previous findings in less severe patients [1,21,33] (Van Dam, Vedel, Ehring, Emmelkamp: Integrated trauma-focused treatment for concurrent posttraumatic stress disorder and substance use disorder: a randomized controlled trial, submitted). There may be several explanations for this phenomenon (Van Dam, Vedel, Ehring, Emmelkamp: Integrated trauma-focused treatment for concurrent posttraumatic stress disorder and substance use disorder: a randomized controlled trial, submitted). First, SUD treatment was equal in both conditions, which may have been so effective that group differences were leveled out. In addition, long-term follow-up may be needed to prove differences between the two conditions on SUD outcomes; PTSD improvements have a better chance to positively influence SUD symptoms after a longer period of time [21] (Van Dam, Vedel, Ehring, Emmelkamp: Integrated trauma-focused treatment for concurrent posttraumatic stress disorder and substance use disorder: a randomized controlled trial, submitted). A 1-year follow-up assessment is currently underway.

Besides the small sample size, a number of additional limitations are noteworthy. First the current sample comprised a mixed group of inpatients and day-care patients. However, the setting for inpatients and day-care patients was very similar. For example, both groups attended their treatment at the same location, the content of both programs was alike, and most important, the group intervention for SUD was the same in both conditions. Second, patients suffering from borderline personality disorder were excluded from the study due to ethical reasons. It is therefore not clear whether the current results also apply to this subgroup of patients. Third, whereas diagnoses of PTSD and SUD were established using structured clinical interviews at pre- and post-treatment, the 3 month follow-up assessment exclusively comprised self-report measures, which can be regarded as a limitation of the current study. A 1 year follow-up assessment including structured clinical interviews to assess diagnostic criteria is currently underway and will provide more conclusive evidence on the long-term effects of the two treatment conditions.

An important strength of this RCT is its specific focus on external validity. The intervention was studied in a routine clinical setting under everyday circumstances. This means that results can easily be generalized to regular clinical practice. Another strength was that all patients were offered the same type of SUD treatment, facilitating interpretations about the added value of TAU + SWT compared to TAU.

Although the small sample size and the indirect nature of findings, supporting a superiority of SWT + TAU, prevent us from drawing firm conclusions, the outcomes of this study are encouraging enough to continue investigating trauma-focused treatment for patients with concurrent PTSD and SUD. Trauma-focused PTSD treatment preliminary appears more effective in decreasing PTSD and SUD symptoms than SUD treatment alone, without jeopardizing patient’s safety or treatment retention [33,34], also if it concerns a more severe SUD patient group.

## Endnotes

^a^Fisher’s excact test was calculated with integers.

^b^Fisher’s excact test was calculated with integers.

## Competing interests

The authors declare that they have no competing interests.

## Authors’ contributions

PE contributed to the writing of the research proposal, participated in the design of the study, supervised the research therapists, supervised data-collection processes, and read and reviewed the manuscript. EV contributed to the writing of the research proposal, participated in the design of the study, supervised data-collection processes, and read and reviewed the manuscript. TE carried out the submission of the research to the ethics committee, participated in the design of the study, supervised and participated in data-collection, supervised data analyses, and read and reviewed the manuscript. DD contributed to the study design, carried out and supervised participant recruitment, inclusion and measurements, carried out data analyses, and contributed to the writing of the research manuscript. All authors read and approved the final manuscript.

## Pre-publication history

The pre-publication history for this paper can be accessed here:

http://www.biomedcentral.com/1471-244X/13/172/prepub
